# Recent Advances in Plant Metabolomics: From Metabolic Pathways to Health Impact

**DOI:** 10.3390/biology11020238

**Published:** 2022-02-02

**Authors:** Andreia Figueiredo, Philippe Hugueney, Alessandra Durazzo

**Affiliations:** 1 Grapevine Pathogen Systems Lab (GPS Lab), Biosystems & Integrative Sciences Institute (BioISI), Faculdade de Ciências da Universidade de Lisboa, 1749-016 Lisboa, Portugal; 2 Santé de la Vigne et Qualité du Vin, INRAE, Université de Strasbourg, 68000 Colmar, France; 3 CREA-Research Centre for Food and Nutrition, Via Ardeatina, 546, 00178 Rome, Italy

In the past decade, technological development allowed a rapid advance on several OMIC approaches, metabolomics was no exception. Advances on the detection and quantification of hundreds of metabolites established metabolomics as one of the most promising areas in both basic and applied studies [1,2]. Within the different biological models, plants are considered to be the richest in metabolite diversity. Considering both primary and secondary metabolites, over 200,000 metabolites are estimated in the plant kingdom [3]. These metabolites present a wide range of functions from plant growth and development to specialized metabolites that are accumulated in response to stress [3]. In addition, with genomes of many plants being sequenced, functional genomics approaches demand has promoted the development of multi-omics-based strategies, with a growing importance of metabolomics. Plant metabolomics is thus, one of the most challenging areas, with proven impact on crop improvement, bioactive compounds identification, plant development and stress related responses.

The integration of metabolomics with other -omics data, including genomics, transcriptomics, and proteomics, through systems biology approaches provides us with a more complete overview of metabolic network regulation and cellular functions [4]. Advances in the field of metabolomics have contributed significantly toward an understanding of plant biology and identification of key pathways and plant molecules that participate in several biological processes. It also enabled a deeper knowledge on food composition, the development of novel dietary markers and ultimately the establishment of metabolic engineering approaches and the use of plants as bioreactor organisms. Considering human health, plant metabolites are also drawing attention with proven beneficial effects against disease, namely diabetes, hypertension, cancer, among others.

This Biology Special Issue “Recent Advances in Plant Metabolomics: From Metabolic Pathways to Health Impact” addresses cutting-edge knowledge on plants’ metabolism, underlying stress responses, plant natural product chemistry and benefits and the role of plant metabolites in human nutrition and health. It comprises 4 articles from 26 authors [5,6,7,8] that mainly summarized the status, applications, and challenges of plant metabolomics in the context of crop breeding, food quality and safety, and human nutrition and health. It also addresses the impact of plant metabolites such as chicoric and rosmarinic on the toxicity of thiacloprid, one of the main insecticides used in agriculture to control pests [8].

Neonicotinoids are one of the most commercially used class of insecticides. They are selective agonists of insect nicotinic acetylcholine receptors (*n*AChRs) [9]. Thiacloprid (TH) is a neonicotinoid compound widely used in agriculture, mainly for foliar application. TH is reported to be ecologically benign however, several reports, including the study by Farag and co-workers [8] show that it presents a negative impact on vertebrates and possibly on humans. Farag et al. [8] explored the effect of chicoric and rosmarinic acids on the insecticide induced-toxicity in chicken embryos. A correlation between the toxicity of TH dose and oxidative damage in the brain of exposed embryos was shown by the induction of oxidative stress, inflammatory response, and the altered expressions of apoptotic and stress-related genes. Moreover, these authors have highlighted the properties of chicoric and rosmarinic acids as anti-apoptotic and anti-reactive oxygen species (ROS)-mediated damages by enhancing brain antioxidants and reducing cytokines and inflammatory mediators. They have shown that combining chicoric and rosmarinic acids presents a powerful natural antioxidant activity against neonicotinoids-induced oxidative injuries [8]

When considering human nutrition and crop improvement, the review by Sun et al. [5] highlights metabolomics as an important approach to understand plant’s chemical complexity in tissues, organs, developmental stages or in stress conditions, as well as to assess food security. Instrumentation for metabolomics-based studies is discussed bringing attention to the fact that no single metabolomics method can determine all metabolites in a sample and that different analytical approaches should be considered to have the best possible coverage of the metabolome [5]. The selection of the metabolomics approach (targeted vs. non-targeted) as well as the pipeline established, were pointed to be crucial and fully dependent on experiment aims. The need for standardization of plant metabolomics studies was also brought into the discussion. Then, Sun and co-workers [5] highlight the enormous potential for crop improvement of linking specific metabolites or metabolic pathways with health and nutrition-related traits. In that sense, metabolite quantitative trait loci (mQTLs) and metabolome-based genome-wide association study (mGWAS) comes to the front of the application of metabolomics in breeding programs [5]. On the following section, authors focus on rice, maize, soybean, wheat, and other crops applications of metabolomics, also highlighting its impact on the assessment of quality and metabolite variation of plant-derived products and food safety [5].

Considering the nutritional qualities of fruits, the study by Commisso et al. [6] evaluated the metabolic diversity and antioxidant activity of six false fruits of apple (*Malus domestica*) and five pear (*Pyrus communis*) cultivars, as tools to identify varieties with superior organoleptic properties and potential health benefits. Authors used both untargeted and targeted metabolomic approaches combining different techniques: nuclear magnetic resonance (NMR) spectroscopy, high-performance liquid chromatography with diode array detection (HPLC-DAD) and HPLC with electrospray ionization mass spectrometry (HPLC-ESI-MS). Results allowed the correlation between antioxidant capacity of the extracts (FRAP assay) and the presence of specific metabolites. This study highlighted one ancient Italian apple cultivar to be rich in polyphenols and one pear cultivar to be low in sucrose, traits that may be particularly attractive to consumers [6].

Considering human use of plants, tea comes as one of the most widely consumed beverages worldwide. One of the main plants used for tea is *Camellia sinensis* (L.) Kuntze. Although the metabolic composition of tea plant leaves has been widely studied, few studies focus on metabolite modulation in response to stress. *Camellia sinensis* trees may freeze to death during overwintering every year, needing replantation and inducing elevated costs. In the study of Wu et al. [7], transcriptome and metabolome analysis were used to examine the freezing resistance mechanism of 60-year-old *C. sinensis* trees under natural freezing stress. Plant samples were compared in two conditions, extreme temperature (average −10 °C) and control samples (average temperature of 15 °C). Metabolomics followed an untargeted approach by HPLC-MS/MS. Over 10,000 differentially accumulated metabolites were found, of which 373 were assigned to 171 KEGG functional categories. Pathway analysis showed that the phenylpropanoid pathway was promoted as well as both carbohydrate and fatty acid pathways [7].

All these studies point out to the tremendous potential of metabolomics, even if this is still considered as an emerging omics approach. With the continuous optimization and improvement of analytical equipment and analysis methods, metabolomics will be even more accurate and comprehensive, allowing us to gain deeper insight into the of intricate and complex plant metabolism.

## Data Availability

Not applicable.
